# Visuospatial cognition in acute unilateral peripheral vestibulopathy

**DOI:** 10.3389/fneur.2023.1230495

**Published:** 2023-09-14

**Authors:** Sun-Young Oh, Thanh Tin Nguyen, Jin-Ju Kang, Valerie Kirsch, Rainer Boegle, Ji-Soo Kim, Marianne Dieterich

**Affiliations:** ^1^Jeonbuk National University College of Medicine, Jeonju, Republic of Korea; ^2^Department of Neurology, Jeonbuk National University Hospital & School of Medicine, Jeonju, Republic of Korea; ^3^Research Institute of Clinical Medicine of Jeonbuk National University-Biomedical Research Institute of Jeonbuk National University Hospital, Jeonju, Republic of Korea; ^4^Department of Pharmacology, Hue University of Medicine and Pharmacy, Hue University, Hue, Vietnam; ^5^Department of Neurology, Ludwig-Maximilians-University, Munich, Germany; ^6^German Center for Vertigo and Balance Disorders, Ludwig-Maximilians-University, Munich, Germany; ^7^Department of Neurology, Seoul National University Bundang Hospital & School of Medicine, Seoul, Republic of Korea; ^8^Munich Cluster for Systems Neurology (SyNergy), Munich, Germany

**Keywords:** acute unilateral peripheral vestibulopathy, vestibular neuritis, vestibular compensation, visuospatial cognition, higher vestibular cognition, vestibular dominance

## Abstract

**Background:**

This study aims to investigate the presence of spatial cognitive impairments in patients with acute unilateral peripheral vestibulopathy (vestibular neuritis, AUPV) during both the acute phase and the recovery phase.

**Methods:**

A total of 72 AUPV patients (37 with right-sided AUPV and 35 with left-sided AUPV; aged 34–80 years, median 60.5; 39 males, 54.2%) and 35 healthy controls (HCs; aged 43–75 years, median 59; 20 males, 57.1%) participated in the study. Patients underwent comprehensive neurotological assessments, including video-oculography, video head impulse and caloric tests, ocular and cervical vestibular-evoked myogenic potentials, and pure-tone audiometry. Additionally, the Visual Object and Space Perception (VOSP) battery was used to evaluate visuospatial perception, while the Block design test and Corsi block-tapping test assessed visuospatial memory within the first 2 days (acute phase) and 4 weeks after symptom onset (recovery phase).

**Results:**

Although AUPV patients were able to successfully perform visuospatial perception tasks within normal parameters, they demonstrated statistically worse performance on the visuospatial memory tests compared to HCs during the acute phase. When comparing right versus left AUPV groups, significant decreased scores in visuospatial perception and memory were observed in the right AUPV group relative to the left AUPV group. In the recovery phase, patients showed substantial improvements even in these previously diminished visuospatial cognitive performances.

**Conclusion:**

AUPV patients showed different spatial cognition responses, like spatial memory, depending on the affected ear, improving with vestibular compensation over time. We advocate both objective and subjective visuospatial assessments and the development of tests to detect potential cognitive deficits after unilateral vestibular impairments.

## Introduction

The spatial cognitive process is the result of complex multisensory signal interactions as well as various delicate synaptic integrative mechanisms involved in cognitive mapping (1, 2). The contribution of vestibular inputs to spatial cognition has been demonstrated in several neurophysiological, neuroimaging, and neuropathological studies (3). The vestibular system participates in dynamic mechanisms of spatial cognition, such as path integration, and landmark-or geometry-based strategies (1, 4), and contributes to update the navigator’s current position in relation to a reference point, space knowledge of the navigable space geometry, recognition of familiar view-dependent scenes, differentiation of self-or object-motion, and optimization of distance estimation (4). Peripheral vestibular signals in convergence with other sensory inputs establish multisensory pathways for enhanced perception and effective navigation (5, 6). Vestibular signals project into many subcortical and cortical structures responsible for spatial cognition, including the thalamocortical and cerebellocortical pathways linking the head direction cells (7).

The causal association between vestibular impairments and visuospatial cognitive deficits was demonstrated in bilateral vestibulopathy (BVP), which provided a comprehensive description of severe and prolonged dysfunction of spatial cognition with hippocampal atrophy (8). Acute unilateral peripheral vestibulopathy (AUPV, most commonly vestibular neuritis) refers to a sudden loss of ipsilateral peripheral vestibular function without hearing impairment or brainstem signs (9). It is characterized by acute, prolonged spontaneous vertigo, nausea/vomiting, and unsteadiness of stance and gait. The symptoms subside within a few weeks (9). Neurotological examinations reveal spontaneous horizontal-torsional nystagmus beating away from the lesion side, an abnormal head impulse test for the involved semicircular canals, and ipsilesional caloric paresis. The vestibular compensation following AUPV was elucidated by virtue of advances in neuroimaging, such as positron emission tomography (PET) (10) and voxel-based morphometry (VBM) on MRI (11). To date, convincing evidence for visuospatial cognitive deficits is lacking in AUPV. Only a few animal studies on AUPV reported spatial cognitive impairments, particularly transient memory deficits within 2 weeks after labyrinthectomy (12), with lesions on the vestibular dominant side suspected of causing more severe cognitive deficits (13). Similarly, several studies showed spatial cognitive deficits (14) and hippocampal atrophy in patients with unilateral vestibulopathy (15). However, other studies failed to confirm these findings (16). The extent of unilateral or bilateral vestibular damage may explain these controversial results because patients often present with incomplete vestibular damage. In several neuroimaging studies, brain metabolism differed between patients with left-and right-sided AUPV, which may explain why the lesions on the dominant side of vestibular lateralization cause more severe spatial cognitive deficits (17). These findings raise questions about the hypothesis that AUPV is associated with spatial cognitive dysfunction, both during the acute stage and after symptoms have resolved in the recovery phase.

Here, we conducted a detailed study of spatial cognition in AUPV patients by using variable visuospatial-perception and-memory tasks. The main purpose of current study was to evaluate the spatial cognitive deficits, visuospatial perception and memory, in patients with AUPV during the initial and recovery phases of vestibular dysfunction. We hypothesized that patients with AUPV would present spatial cognitive deficits during the acute phase, especially when the right side, the dominant vestibular side in right-handers, is affected.

## Methods

### Participants

The study included 72 AUPV patients (37 right and 35 left AUPV; age range 34 to 80 years, median 60.5; 39 men, 54.2%) and 35 healthy controls (HCs; age range 43 to 75 years, median 59; 20 men, 57.1%) in Jeonbuk National University Hospital from March 2021 to August 2022 (Table 1). Patients who had moderate to severe visual impairment (with a best-corrected visual acuity less than 6/18 in the better eye) (18) or hearing impairment (with a threshold of pure tone audiometry over 30 dB), clinical signs of central involvement (such as gaze-evoked nystagmus, skew deviation, associated neurological deficits), abnormal MRI findings with diffusion-weighted sequence, or were on centrally active medications or vestibular sedatives were excluded. For all participants, the Mini-Mental State Examination (MMSE) was used to assess global mental status (19), and the Edinburgh Handedness Inventory, a 10-item inventory, to assess handedness (Table 1). During the acute phase, the visual analog scale (VAS) for dizziness (D-VAS) was used to gage the subjective feeling of dizziness in AUVP patients. Patients were asked about their personal experience of dizziness, which they reported using the D-VAS. The scale ranged from 0 (indicating no sensation) to 100 (representing a disabling and continuous sensation). The Visual Object and Space Perception (VOSP) battery was used to assess visuospatial perception, and the Block design test (BDT) and Corsi block-tapping test (CBTT) were performed to assess visuospatial memory (Table 2).

**Table 1:** Comparison of demographic features and vestibular function tests in AUPV (vestibular neuritis, VN) patients (*n* = 72) and healthy controls (*n* = 35).

	VN (*n* = 72)	R.VN (*n* = 37)	L.VN (*n* = 35)	HC (*n* = 35)	Value of *p* (VN-HC)	Value of *p* (R.VN-HC)	Value of *p* (L.VN-HC)	Value of *p* (R.VN-L.VN)
Demographics								
Sex, male, *n* (%)	39 (54.17)	20 (54.05)	19 (54.29)	20 (57.1)	0.758	0.777	0.793	0.984
Age, years, median (95% CI)	60.5 (59–63)	62 (58–65)	60 (58–63)	59 (53–65)	0.421	0.252	0.78	0.379
Education, years, median (95% CI)	12 (12–16)	12 (12–16)	12 (12–16)	16 (11–16)	0.474	0.503	0.539	0.9
MMSE (30 points), median (95% CI)	28 (28–29)	28 (28–29)	29 (28–30)	28 (28–30)	0.581	0.417	0.87	0.455
Right handedness, *n* (%)	72 (100)	37 (100)	35 (100)	35 (100)				
D-VAS	39.95 ± 1.75	41.01 ± 1.07	37.24 ± 1.90	-				0.591
Audio-Vestibular function tests								
Acute phase (within 2 days of onset)								
Spontaneous nystagmus, mean (°/sec)	13.7 ± 14.5	14.2 ± 11.3	12.9 ± 14.1	-	-			0.061
vHIT hVOR mean gain								
Ipsilesional, median (95% CI)	0.69 (0.61–0.76)	0.64 (0.53–0.83)	0.72 (0.62–0.76)	-	-			0.968
Contralesional, median (95% CI)	0.96 (0.94–0.99)	0.94 (0.91–0.98)	0.97 (0.96–1.01)	-	-			0.178
Presence of corrective saccades, *n* (%)	58 (80.56)	28 (75.68)	30 (85.71)	-	-			0.285
Caloric paresis, %, median (95% CI)	66.96 (45.7–98)	68.8 (36.4–110)	66.96 (36.4–111)	-	-			0.782
Caloric paresis ≥35%, *n* (%)	48 (66.67)	25 (67.57)	23 (65.71)	-	-			0.933
Cervical and ocular VEMP								
cVEMP p13 mean latency								
Ipsilateral, ms, median (95% CI)	13.9 (13.6–14.6)	13.9 (13.6–14.4)	14.2 (13.5–15.3)	-	-			0.515
Contralateral, ms, median (95% CI)	13.9 (13.5–14.2)	13.6 (13.2–14.2)	14.1 (13.2–14.6)	-	-			0.48
cVEMP amplitude AR, %, median (95% CI)	21 (13–26)	16 (12–25)	25 (15–31)	-	-			0.28
cVEMP amplitude AR ≥ 40%, *n* (%)	11 (15.28)	4 (10.81)	7 (20)	-	-			0.116
oVEMP n10 mean latency								
Ipsilateral, ms, median (95% CI)	11 (10.8–11.8)	11 (10.7–11.8)	11.3 (10.8–12.8)	-	-			0.118
Contralateral, ms, median (95% CI)	10.7 (10.4–10.8)	10.7 (10.3–10.8)	10.55 (10.2–11)	-	-			0.624
oVEMP amplitude AR, %, median (95% CI)	26 (17–36)	21.5 (14–45)	27 (14–44)	-	-			0.925
oVEMP amplitude AR ≥ 40%, *n* (%)	20 (27.78)	11 (29.73)	9 (25.71)	-	-			0.855
PTA, dB, median (95% CI)	19.5 (15–24)	20.5 (16–24.5)	15.8 (12.5–24.5)	-	-			0.396
Recovery phase (follow-up 4 weeks after onset)								
vHIT hVOR mean gain					-			
Ipsilesional, median (95% CI)	0.82 (0.57–1.02)	0.85 (0.57–1.03)	0.76 (0.53–1.06)	-	-			0.48
Contralesional, median (95% CI)	0.97 (0.93–1.03)	0.96 (0.91–1.01)	0.99 (0.91–1.07)	-	-			0.41
Presence of corrective saccades, *n* (%)	22 (30.56%)	9 (24.3%)	13 (37.1%)					0.307

Values are presented as median (95% CI). Statistical significance was calculated using the Mann–Whitney U test. D-VAS, the visual analog scale (VAS) for dizziness; vHIT-ipsi, video head impulse test-ipsilesional; UW, unilateral weakness; VEMP, vestibular evoked myogenic potential; AR, asymmetry ratio; MMSE, mini–mental state examination; PTA, pure tone audiometry; dB, decibel; ms, millisecond.

**Table 2:** Assessment of visuospatial cognitive abilities in AUPV (vestibular neuritis, VN) patients during acute and recovery phases.

	VN (*n* = 72)	R.VN (*n* = 37)	L.VN (*n* = 35)	HC (*n* = 35)	Value of *p* between group^K^	Value of *p*^M^ (VN-HC)	Value of *p*^M^ (R.VN-HC)	Value of *p*^M^ (L.VN-HC)	Value of *p*^M^ (R.VN-L.VN)
Acute phase (within 2 days of onset)									
Visuospatial perception tests									
Position discrimination (20 points)	18 (18–19)	18 (17–19)	19 (18–20)	20 (19–20)	**0.003**	**0.006**	**0.001**	0.083	0.042
Number location (10 points)	9 (8–9)	8 (8–9)	9 (9–10)	10 (9–10)	**<0.001**	**<0.001**	**<0.001**	0.023	**0.006**
Cube analysis (10 points)	9 (8–9)	8 (8–9)	10 (9–10)	9 (9–10)	**<0.001**	0.048	**<0.001**	0.834	**<0.001**
Visuospatial memory tests									
BDT (48 points)	32 (32–32)	30 (28–32)	32 (32–36)	37 (32–40)	**<0.001**	**0.002**	**<0.001**	0.095	**0.003**
BDT Plus (66 points)	34 (32–35)	33 (29–34)	35 (34–41)	43 (38–48)	**<0.001**	**0.001**	**<0.001**	0.041	0.019
CBTT-block span	5 (5–6)	5 (5–6)	6 (5–6)	7.5 (7–8)	**<0.001**	**<0.001**	**<0.001**	**<0.001**	0.098
CBTT-total score	25 (23–28)	24 (21–28)	27 (22–33)	40.5 (34–44)	**<0.001**	**<0.001**	**<0.001**	**<0.001**	0.047
Recovery phase (follow-up 4 weeks after onset)									
Visuospatial perception tests									
Position discrimination (20 points)	19 (19–20)	19 (19–20)	20 (19–20)	20 (19–20)	0.488	0.708	0.402	0.868	0.259
Number location (10 points)	9 (9–10)	9 (9–10)	10 (9–10)	10 (9–10)	0.138	0.353	0.091	0.983	0.094
Cube analysis (10 points)	9 (9–10)	9 (9–10)	9 (9–10)	9 (9–10)	0.289	0.362	0.17	0.793	0.205
Visuospatial memory tests									
BDT (48 points)	32 (32–36)	32 (32–38)	32 (32–38)	37 (32–40)	0.155	0.065	0.075	0.131	0.528
BDT Plus (66 points)	38 (35–40)	36 (34–41)	38 (35–47)	43 (38–48)	0.097	0.067	0.037	0.231	0.236
CBTT-block span	7 (6–7)	7 (6–7)	7 (6–8)	7 (7–8)	0.187	0.099	0.062	0.272	0.446
CBTT-total score	35.5 (31–38)	35 (29–36)	37 (31–43)	40.5 (34–44)	**0.037**	0.051	**0.012**	0.308	0.1

Values are presented as median (95% CI). Statistical significance was calculated using the ^K^Kruskal-Wallis test (between group comparison) and the ^M^Mann–Whitney U test (pairwise comparisons) with a Bonferroni-adjusted significance level of 0.017 (0.05/3). Bold indicates a statistically significant difference. In the BDT, scores are added based on whether the block design for each question is correct regardless of time, and the BDT Plus is a summation assigning additional points for faster answers. CBTT-block span is the length of the last correctly repeated sequence. CBTT-total score is the product of the CBTT-block span and the number of correctly repeated sequences until the test is discontinued (i.e., the number of correct trials). This latter score takes into account the performance on both trials of an equal length and is more reliable than the CBTT-block span alone.

All participants provided informed consent and received monetary compensation for participation. The Institutional Review Board at Jeonbuk National University Hospital (no. 2020-10-134-006) reviewed and approved the experiments.

### Vestibular function tests

All patients underwent neurotological investigations using video-oculography, the video head impulse test (vHIT) and caloric test, ocular and cervical vestibular-evoked myogenic potentials (VEMPs), and pure-tone audiometry within the first 2 days (acute phase) and 4 weeks after symptom onset (recovery phase). vHIT was performed more than 20 times (head rotation 15–20°, duration 150–200 ms, peak velocity > 150°/s) on both sides of each plane and was analyzed using oculography (SLMED, Seoul, Korea) (20). The caloric irrigation test was performed with the patient in the supine position and 30^0^ head elevation using closed-loop water irrigators at 30°C and 44°C (irrigation time 30 s, intervals 5 min) and was characterized by induced nystagmus (SLMED, Seoul, Korea) (21), especially the slow-phase velocity to estimate unilateral weakness using Jongkees formula (22). For cervical VEMPs (23), active electrodes were placed over the middle or upper portion of the sternocleidomastoid muscle; for ocular VEMPs (24), electrodes were placed on the infraorbital margin 1 cm below the center of the contralateral lower eyelid. The VEMP results can be easily interpreted based on the asymmetry ratio (AR) of the amplitude, computed as the difference in amplitudes between the ears divided by the sum of the amplitudes in both ears (25).

### Visuospatial perception testing (VOSP battery)

The Visual Object and Space Perception (VOSP) battery is a neuropsychological assessment tool used to evaluate visual perception and spatial processing abilities. It includes subtests for Object Perception and Space Perceptions, which are designed to elicit straightforward responses from participants and minimize the influence of other cognitive abilities (26, 27). In this study, we first conducted a preliminary visual sensory efficiency test (Shape detection screening test), and then administered the Space Perceptions subtests, including Position discrimination, Number location, and Cube analysis.

*The shape detection screening test* was conducted to ensure that participants had adequate visual capacity to complete the other subtests. Visual acuity of the participants was assessed using 20 stimulus cards, half of which contained a degraded ‘X’ symbol (degraded by 30%), and participants were required to identify the presence or absence of the ‘X’ (28). Participants with scores of 15 or below were excluded from further participation in the VOSP test battery, as research has shown that low visual acuity can significantly affect performance on the VOSP tasks (28).

*The position discrimination test* includes 20 boards, each featuring a square with a black dot (5 mm) positioned exactly at the center, and another square with a slightly off-center black dot that is horizontally adjacent. The score is determined by counting the number of correct responses in identifying the square with the black dot at the exact center, with a maximum possible score of 20 (20). The cutoff value for failure is 18/20 (28).

*The number location test* comprises of 10 boards, each containing two squares with a small gap between them. The top square displays randomly arranged numbers (1–9), while the bottom square has a single black dot that corresponds to the position of one of the numbers. The score is based on the number of correct responses identifying the number that matches the dot’s position, with a maximum score of 10 (20). The cutoff value for failure is 7/10 (28).

*The cube analysis test* is a three-dimensional (3D) analysis presented on a two-dimensional (2D) plane consisting of 10 boards with 3D-arranged cubes. The score is determined based on the number of correct responses accurately identifying the number of cubes were on each board, including the hidden cube (maximum score: 10) (20). The cutoff value for failure is 6/10 (28).

### Visuospatial memory testing

#### Block design test

Participants were given nine individual blocks with two sides of solid white, two sides of solid red, and two sides of half red/half white (crossed diagonally) and were asked to assemble the blocks to exactly reconstruct the 2D pattern shown (29). Gradually more complex patterns are presented and reproduction times are measured. Each trial is timed and bonus points are given for faster completion. BDT scores range from 0 to 48, with bonus points up to 66 (BDT Plus). BDT is considered to reflect spatiotemporal structural capabilities and is a reasonably good predictor for routine spatial measurements (30). A higher score reflects better visuospatial functioning.

#### Corsi block-tapping test

The examiner tapped cubes starting with a sequence of two blocks in front of the participant. Two trials were performed per block sequence length. The participant had to tap the cube sequence in the same order immediately after the examiner had finished. The number of cubes tapped ranged from 2 to 9. The subject had two chances to tap the cubes in the correct order; the subject only proceeded to the next step if he or she provided the correct answer (20). For each patient, the two metrics block span and total score were measured. The CBTT-block span is equal to the length of the last correctly repeated sequence. The CBTT-total score is the product of the block span and the number of correctly repeated sequences during the test. Considering the performance on both trials of equal length, the CBTT-total score is more accurate (31). The CBTT is a simple and effective method to assess visuospatial working memory and spatial attention.

### Statistical analysis

All data were analyzed using SPSS Statistics version 23.0 (IBM Corp., Armonk, NY, USA). Nonparametric variables are displayed as median values accompanied by a 95% confidence interval (CI), whereas parametric variables are shown as the mean ± standard deviation (SD). Frequencies are represented by counts and their respective percentages. To assess statistical significance, the Kruskal-Wallis test was utilized for comparisons between groups, while the Mann–Whitney U test was employed for pairwise comparisons. For each subgroup, comparisons between acute and recovery phases were assessed using the Wilcoxon Signed Rank test. A value of *p* less than 0.05 and a Bonferroni-adjusted significance level of 0.017 (0.05/3) was considered statistically significant for pairwise comparisons within the three groups.

## Results

### Demographics and clinical data

The demographic and clinical characteristics of the patients are summarized in Table 1. The patients had a median education of 12 years (95% CI: 12–16) and maintained an overall cognitive function, as indicated by a median MMSE score of 28 (95%CI: 28–29). No significant differences were observed in baseline education levels and general cognitive abilities (MMSE) between AUPV patients and HCs, as assessed by the Mann–Whitney U test. Furthermore, the shape detection screening test confirmed that patients had sufficient visual capacity to undertake the other subtests and demonstrated no significant variations between AUPV patients and the HC group in the current study. All participants in the study were identified as right-handed. AUPV patients were categorized according to the affected ear into either right (*n* = 37) or left (*n* = 35) subgroups. Importantly, there were no notable demographic differences between these subgroups. During the acute phase, both groups exhibited comparable levels in general cognition, sex distribution, education, and visual capability. Moreover, the D-VAS scores, which evaluate the subjective feeling of dizziness in AUPV patients, did not show any significant difference between the right VN and left VN groups. This is another factor to consider that during the acute phase of AUPV, patients’ sensations of dizziness might have influenced their performance on visuospatial attention and memory tasks.

The patients with AUPV (vestibular neuritis) mostly presented with acute or subacute spontaneous vertigo with nausea, vomiting, and unsteadiness. Vertigo was usually described as rotational and markedly increased with head position changes. On first examination, spontaneous nystagmus was directed to the contralesional side with a mean slow phase velocity of 13.7°/s (± 14.5) in the patient group, which was similar between the right and left AUPV subgroups (14.2 ± 11.3°/s vs. 12.9 ± 14.1°/s, *p* = 0.061). Almost all patients showed pathological findings on bedside HIT, and the median caloric weakness value was 66.96% (95%CI: 45.7–98%) in the patient group. The vHIT gain was decreased with a mean value of 0.69 (95%CI: 0.61–0.76) for the ipsilesional side and within normal range with a mean of 0.96 (95%CI: 0.94–0.99) for the contralesional side; corrective saccades were mostly observed on the ipsilesional side. In the AUPV group, the average pure tone audiometry value was 19.5 dB (95% CI: 15–24), which demonstrates normal hearing capabilities. The AR of cervical VEMP amplitudes was 21% (median, 95%CI: 13–26%), with abnormal AR (>40%) in 15.28% (11/72). The AR of ocular VEMP amplitudes was 26% (median, 95%CI: 17–36%), with abnormal AR found in 27.78% (20/72). Significant differences were not observed in the vestibular function tests between the right and left AUPV subgroups (Table 1).

### Visuospatial cognition during the acute phase of AUPV

**Figure 1:**
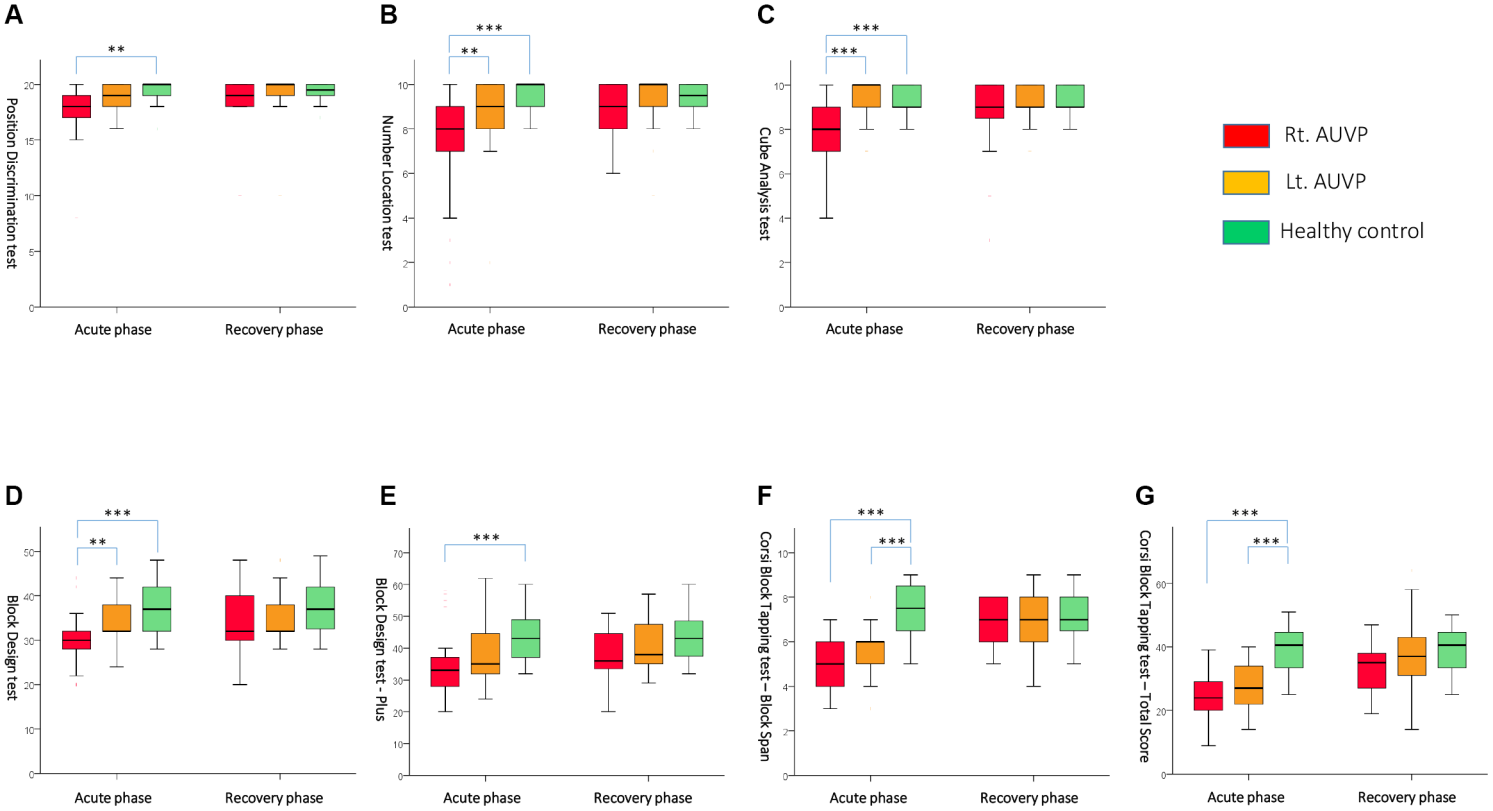
Comparisons of visuospatial cognitive performances between subgroups during the acute and recovery phases with **(A)** Position Discrimination test, **(B)** Number Location test, **(C)** Cube Analysis test, **(D)** Block Design test, **(E)** Block Design test-Plus, **(F)** Corsi Block Tapping test-Block Span, and **(G)** Corsi Block Tapping test-Total Score. Rt. AUVP, right-sided acute unilateral vestibulopathy; Lt. AUVP, left-sided acute unilateral vestibulopathy. **Indicates *p* < 0.01; ***indicates *p* < 0.001. Statistical significance was calculated using the Mann–Whitney U test with a Bonferroni-adjusted significance level of 0.017 (0.05/3).

During the acute phase, the AUPV group displayed impairments in visuospatial perception and memory tests compared to the HCs, as shown in Table 2. In the visuospatial perception test, the AUPV patients scored significantly lower in Position discrimination (18 vs. 20, *p* = 0.006, the Mann–Whitney U test), Number location (9 vs. 10, *p* < 0.001), and Cube analysis (9 vs. 10, *p* = 0.048) compared to HCs. However, despite these lower scores, all values surpassed the cutoff values for failure, which are 18, 7, and 6, respectively. In the visuospatial memory test, the AUPV patients demonstrated significantly lower scores in the Block design tests (BDT, 32 vs. 37, *p* = 0.002; BDT Plus, 34 vs. 43, *p* = 0.001) and CBTT tests (block span, 5 vs. 7.5, *p* < 0.001; total score, 25 vs. 40.5, *p* < 0.001) compared to HCs, as assessed by the Mann–Whitney U test (Figure 1).

In a subgroup analysis comparing right and left AUPV patients, the right AUPV group scored significantly lower in visuospatial perception (Position discrimination, 18 vs. 19, *p* = 0.042; Number location, 8 vs. 9, *p* = 0.006; Cube analysis, 8 vs. 10, *p* < 0.001) and visuospatial memory tests (BDT, 30 vs. 32, *p* = 0.003; BDT Plus, 33 vs. 35, *p* = 0.019; CBTT-total score, 24 vs. 27, *p* = 0.047) according to the Mann–Whitney U test (Table 2). Compared to the HC group, the right AUPV group had also significantly lower scores in both visuospatial perception (Position discrimination, 18 vs. 20, *p* = 0.001; Number location, 8 vs. 10, *p* < 0.001; Cube analysis, 8 vs. 9, *p* < 0.001) and memory (BDT, 30 vs. 37, *p* < 0.001; BDT Plus, 33 vs. 43, *p* < 0.001; CBTT-block span, 5 vs. 7.5, *p* < 0.001; CBTT-total score, 24 vs. 40.5, *p* < 0.001) as evaluated by the Mann–Whitney U test. The left AUPV subgroup exhibited a lesser degree of impairment, with significantly lower scores in the Number location test (9 vs. 10, *p* = 0.023), BDT Plus (35 vs. 43, *p* < 0.001), and CBTT (block span, 5 vs. 7.5, *p* < 0.001; total score, 24 vs. 40.5, *p* < 0.001) relative to the HCs, as assessed by the Mann–Whitney U test (Figure 1).

Correlation analysis between the vestibular function tests of ipsilesional vHIT gain and caloric weakness, asymmetry ratio of cervical and ocular VEMP, and the visuospatial cognition tests did not show significant relationships (Table 3, Spearman’s correlation).

**Table 3:** Spearman’s correlation analysis of vestibular function tests and visuospatial cognition parameters.

	vHIT-ipsi HC-gain		Caloric UW		Cervical VEMP AR		Ocular VEMP AR		PTA	
	*r*	*p*	*r*	*p*	*r*	*p*	*r*	*p*	*r*	*p*
Position discrimination	−0.089	0.459	−0.071	0.568	−0.195	0.113	0.24	0.069	−0.045	0.714
Number location	−0.035	0.771	−0.135	0.276	−0.076	0.543	−0.001	0.996	−0.116	0.344
Cube analysis	0.117	0.332	−0.06	0.628	0.205	0.096	0.083	0.534	−0.181	0.137
BDT	0.086	0.478	−0.142	0.251	−0.039	0.755	−0.045	0.735	−0.17	0.162
BDT Plus	−0.047	0.7	−0.116	0.35	−0.056	0.655	−0.051	0.703	−0.239	0.058
CBTT-block span	−0.037	0.768	0.047	0.716	−0.019	0.88	−0.033	0.81	−0.175	0.168
CBTT-total score	−0.111	0.374	0.025	0.845	−0.085	0.507	−0.1	0.472	−0.186	0.14

vHIT-ipsi, video head impulse test-ipsilesional; HC, horizontal semicircular canal; UW, unilateral weakness; VEMP, vestibular evoked myogenic potential; AR, asymmetry ratio; PTA, pure tone audiometry. The correlations between vestibular function tests and visuospatial cognition parameters were assessed using Spearman’s nonparametric bivariate correlation.

### Visuospatial cognition during the recovery phase of AUPV

Most AUPV patients experienced significant improvement in severe vertigo and static vestibular imbalance within a few days, which continued to resolve over the subsequent weeks. Four weeks after the onset of symptoms, all patients showed recovery from initial symptoms such as vertigo, imbalance, spontaneous nystagmus, and abnormal vestibulo-ocular reflex (VOR) gain (Table 1). Alongside the vestibular compensation process, visuospatial cognitive deficits also improved, resulting in AUPV patients’ scores being comparable to those of the HCs (Tables 2, 4; Figure 1). However, subgroup analysis indicated that the right AUPV group still had significantly lower scores in the visuospatial memory tests with BDT Plus (36 vs. 43, *p* = 0.039) and CBTT-total score (35 vs. 40.5, *p* = 0.019) compared to the HC group, as assessed by the Mann–Whitney U test (Tables 2, 4; Figure 1).

**Table 4:** A paired test illustrating the time-dependent alterations in visuospatial cognitive parameters.

	VN (*n* = 72)			R.VN (*n* = 37)			L.VN (*n* = 35)			HC (*n* = 35)		
	Acute phase	Recovery phase	Value of *p*	Acute phase	Recovery phase	Value of *p*	Acute phase	Recovery phase	Value of *p*	Acute phase	Recovery phase	Value of *p*
Visuospatial perception tests												
Position discrimination	18 (18–19)	19 (19–20)	**<0.001**	18 (17–19)	19 (19–20)	**<0.001**	19 (18–20)	20 (19–20)	0.055	20 (19–20)	19.5 (19–20)	0.564
Number location	9 (8–9)	9 (9–10)	**<0.001**	8 (8–9)	9 (9–10)	**0.001**	9 (9–10)	10 (9–10)	0.149	10 (9–10)	9.5 (9–10)	1
Cube analysis	9 (8–9)	9 (9–10)	**0.001**	8 (8–9)	9 (9–10)	**<0.001**	10 (9–10)	9 (9–10)	0.484	9 (9–10)	9 (9–10)	0.157
Visuospatial memory tests												
BDT	32 (32–32)	32 (32–36)	**<0.001**	30 (28–32)	32 (32–38)	**0.001**	32 (32–36)	32 (32–38)	0.089	37 (32–40)	37 (33–40)	0.083
BDT Plus	34 (32–35)	38 (35–40)	**<0.001**	33 (29–34)	36 (34–41)	**<0.001**	35 (34–41)	38 (35–47)	0.109	43 (38–48)	43 (38–48)	0.854
CBTT-block span	5 (5–6)	7 (6–7)	**<0.001**	5 (5–6)	7 (6–7)	**<0.001**	6 (5–6)	7 (6–8)	**<0.001**	7.5 (7–8)	7 (7–8)	0.234
CBTT-total score	25 (23–28)	35.5 (31–38)	**<0.001**	24 (21–28)	35 (29–36)	**<0.001**	27 (22–33)	37 (31–43)	**0.001**	40.5 (34–44)	40.5 (34–44)	0.276

Values are presented as median (95% CI). Statistical significance was calculated using the Wilcoxon Signed Rank test. Bold *p*-values indicate significant differences.

## Discussion

Although AUPV patients were able to successfully perform visuospatial perception tasks within normal parameters, the current findings revealed a decline in visuospatial perception and memory compared to HCs in the acute phase. These visuospatial cognitive impairments were more pronounced in the acute stage and gradually improved over the course of 4 weeks. These findings align with previous research that also identified visuospatial cognitive deficits in AUPV patients (32). One possible explanation for these impairments might be abnormalities in the vestibular reflex, such as oscillopsia/nystagmus-induced blurred vision or VOR deficits, or from imbalances in stance and gait due to VSR deficits (33). However, this explanation would predict similar spatial cognitive deficits in both left and right AUPV during acute and recovery phases, which was not supported by the current findings. Nonetheless, during the acute phase, there was no significant difference in the D-VAS scores, a measure used to gage the subjective feeling of dizziness in AUPV patients, between those with right VN and those with left VN. Given that there were no marked differences in general cognition, gender distribution, education level, vestibular imbalance, and subjective feelings of dizziness between the right and left VN groups, a more plausible hypothesis might be that spatial cognitive discrepancies during the early stages of AUPV arise from disrupted vestibular information to the hippocampal formation that negatively impacts the multisensory integration of cognitive mapping.

The results revealed a significant difference in performance between right and left AUPV subgroups compared to each other and the HC group. Specifically, more severe and lasting deficits in visuospatial perception and memory were observed in the right AUPV subgroup (vestibular dominant side) than in the left AUPV subgroup (vestibular non-dominant side) of the right-handed patients. A plausible explanation for these differing impairments could be the initial disruption or absence of peripheral input into the bilateral vestibular cortical network, which features predominantly ipsilateral right-sided pathways from the vestibular nuclei to the parietoinsular core region and a right hemispheric vestibular dominance in right-handers (34, 35). This is consistent with a three-month follow-up study in UVD rats, which demonstrated spatial memory deficits in darkness, suggesting spatial navigation impairments independent of oscillopsia (36). Similarly, previous studies, especially differences according to the gender or lesion side, revealed that spatial cognitive performance appeared substantially poorer in female patients (37), and deficits in spatial memory and navigation were found in right but not in left vestibular loss (37). Regarding vestibular lateralization (38), the unilateral lesions on the vestibular dominant side appeared to show more severe deficits than those on the non-dominant side (38). Neuroimaging data also indicated that brain activity in the acute phase of right-and left-sided AUPV exhibited different compensatory patterns, with more pronounced negative metabolic brain activities with right-sided lesions in right-handed patients (39).

Given the complexity of visuospatial memory tests in contrast to visuospatial perception tasks, the more sophisticated the task, the greater the chance of identifying visuospatial deficiencies. From this point of view AUPV tends to cause more recognizable deficits in visuospatial memory, which involves intricate vestibular processes (40), compared to visuospatial perception which is predominantly influenced by visual information (41). This is in alignment with numerous earlier studies that have focused on identifying and defining the impact of vestibular impairment on visuospatial memory (42). Consequently, there is a need to develop clinical visuospatial behavioral tests that can more sensitively identify these minor alterations across different patient groups.

Vestibular information must ascend to the hippocampus to be integrated with visual and other sensory data pertinent to spatial memory (43, 44). This information has been demonstrated to reach the hippocampal formation, a complex brain structure involved in spatial cognition, through various pathways such as thalamocortical, theta-generating, cerebellocortical, and head direction pathways (7, 45). Furthermore, place cells in the hippocampus, which react to specific locations in the environment, are influenced by vestibular stimulation (33). The vestibular system plays a role in the dynamic processes of spatial cognition, including path integration, landmark-based strategies, and geometry-based strategies (1, 4). As for brain morphological changes related to spatial cognition, there is no definitive neuroimaging evidence of hippocampal atrophy in UVD patients (37). Some studies have reported atrophy in the ipsilateral supramarginal nucleus, postcentral and superior temporal gyrus, MT/V5 area, contralateral thalamus, and mesencephalon tegmentum (11, 46). Other studies of patients who recovered from AUPV showed a significant decrease in the volume of left posterior hippocampus (11). The authors speculated that the relative atrophy was the result of interaction between the diminished vestibular input and the insufficient central compensation to ameliorate all features of unilateral peripheral vestibular loss (11).

Despite the vestibular system’s role in integrating multisensory signals of various ipsilateral and contralateral brain regions, both this study and past animal behavioral studies (12), have indicated that a loss of half of the vestibular afferents causes spatial memory and navigation dysfunction during the acute phase of vestibular damage. The swift recovery of spatial cognitive performance in AUPV patients is due to vestibular compensation and adaptation, which restore the reduced activity in the ipsilateral vestibular nuclei and rebalance activity between both sides. Recent studies that focused on visualizing the relative changes in glucose metabolism (rCGM) found significant asymmetries in the vestibular nuclei complexes and related structures of the vestibulo-cerebellum, thalamus, vestibular cortex, hippocampus, and amygdala during the acute stage of UVD (10). This was followed by a rebalance of rCGM within these structures. Additional research has identified abnormalities in cortical and subcortical activations following AUPV. For instance, in a functional MRI study, significant decreases were observed in resting-state activities of the medial aspect of the superior parietal lobule, posterior cingulate cortex, middle frontal gyrus, middle temporal gyrus, parahippocampal gyrus, anterior cingulate cortex, insular cortex, caudate nucleus, thalamus, and midbrain (47). Thus, central compensation of unilateral peripheral vestibular loss involves numerous structures of the bilateral central vestibular network from the vestibular nuclei complexes to vestibular cortex and hippocampus to improve the different vestibular assignments from vestibulo-ocular reflexes at brain stem level to cognitive tasks like spatial orientation and navigation at subcortical/cortical level.

A limitation of this study is the lower sensitivity of the clinical behavioral tests employed, and the absence of functional imaging-based evidence to support the observed cognitive performance findings. Although we observed no marked differences in general cognition or subjective dizziness, the results do not negate potential general cognitive abilities or attentional deficits. Given the limited sensitivity of the MMSE, our chosen cognitive test, interpretations should be approached with caution. Further research using more sensitive cognitive assessments is warranted. Additionally, the study exclusively involved right-handed patients, indicating a need for further research with left-handed AUPV patients for a comprehensive understanding.

In conclusion, for the first time, we assessed visuospatial perception and memory cognition in AUPV patients during the acute phase and early compensation stages. Specifically, AUPV patients demonstrated varying sensitivities in spatial cognition areas, such as spatial memory, based on the affected ear side, with improvements observed as vestibular compensation progressed in the subsequent weeks. We suggest examining both objective and subjective visuospatial cognitive measures and the development of cognitive behavioral tests capable of discerning and identifying potential visuospatial cognitive deficits that may arise following acute or chronic unilateral vestibular impairments.

## Data availability statement

The original contributions presented in the study are included in the article/supplementary material, further inquiries can be directed to the corresponding author.

## Ethics statement

The studies involving humans were approved by the Institutional Review Board at Jeonbuk National University Hospital (no. 2020-10-134-006) reviewed and approved the experiments. The studies were conducted in accordance with the local legislation and institutional requirements. The participants provided their written informed consent to participate in this study.

## Author contributions

S-YO and MD: study conception and design. S-YO and J-JK: data collection. S-YO and TTN: analysis, interpretation of results, and draft manuscript preparation. All authors reviewed the results and approved the final version of the manuscript.

## Funding

This work was supported by a National Research Foundation of Korea (NRF) grant funded by the Korean government (Ministry of Science and ICT) (No. 2022R1A2B5B01001933), the Fund of the Biomedical Research Institute, Jeonbuk National University Hospital, and the German Foundation for Neurology (Deutsche Stiftung Neurologie for MD).

## Conflict of interest

The authors declare that the research was conducted in the absence of any commercial or financial relationships that could be construed as a potential conflict of interest.

## Publisher’s note

All claims expressed in this article are solely those of the authors and do not necessarily represent those of their affiliated organizations, or those of the publisher, the editors and the reviewers. Any product that may be evaluated in this article, or claim that may be made by its manufacturer, is not guaranteed or endorsed by the publisher.
